# Blueberry and cranberry pangenomes as a resource for future genetic studies and breeding efforts

**DOI:** 10.1093/hr/uhad202

**Published:** 2023-10-10

**Authors:** Alan E Yocca, Adrian Platts, Elizabeth Alger, Scott Teresi, Molla F Mengist, Juliana Benevenuto, Luis Felipe V Ferrão, MacKenzie Jacobs, Michal Babinski, Maria Magallanes-Lundback, Philipp Bayer, Agnieszka Golicz, Jodi L Humann, Dorrie Main, Richard V Espley, David Chagné, Nick W Albert, Sara Montanari, Nicholi Vorsa, James Polashock, Luis Díaz-Garcia, Juan Zalapa, Nahla V Bassil, Patricio R Munoz, Massimo Iorizzo, Patrick P Edger

**Affiliations:** Department of Horticulture, Michigan State University, East Lansing, MI, 48824, United States; Department of Plant Biology, Michigan State University, East Lansing, MI, 48824, United States; Department of Horticulture, Michigan State University, East Lansing, MI, 48824, United States; Department of Plant Biology, Michigan State University, East Lansing, MI, 48824, United States; Department of Horticulture, Michigan State University, East Lansing, MI, 48824, United States; Department of Horticulture, Michigan State University, East Lansing, MI, 48824, United States; Genetics and Genome Sciences, Michigan State University, East Lansing, MI, 48824, United States; Plants for Human Health Institute, North Carolina State University, Kannapolis, NC United States; Horticultural Sciences Department, University of Florida, Gainesville, FL 32611, United States; Horticultural Sciences Department, University of Florida, Gainesville, FL 32611, United States; Department of Horticulture, Michigan State University, East Lansing, MI, 48824, United States; Department of Biochemistry and Molecular Biology, Michigan State University, East Lansing, MI, 48824, United States; Department of Horticulture, Michigan State University, East Lansing, MI, 48824, United States; Department of Horticulture, Michigan State University, East Lansing, MI, 48824, United States; University of Western Australia, Perth 6009Australia; Justus Liebig University, Giessen, 35390Germany; Department of Horticulture, Washington State University, Pullman, WA, 99163, United States; Department of Horticulture, Washington State University, Pullman, WA, 99163, United States; The New Zealand Institute for Plant and Food Research Limited (PFR), Auckland, New Zealand; The New Zealand Institute for Plant and Food Research Limited (PFR), Palmerston, New Zealand; The New Zealand Institute for Plant and Food Research Limited (PFR), Palmerston, New Zealand; The New Zealand Institute for Plant and Food Research Limited (PFR), Motueka, New Zealand; SEBS, Plant Biology, Rutgers University, New Brunswick NJ 01019United States; SEBS, Plant Biology, Rutgers University, New Brunswick NJ 01019United States; Department of Viticulture and Enology, University of California, Davis, Davis, CA 95616, United States; Department of Viticulture and Enology, University of California, Davis, Davis, CA 95616, United States; National Clonal Germplasm Repository, USDA-ARS, Corvallis, OR 97333, United States; Horticultural Sciences Department, University of Florida, Gainesville, FL 32611, United States; Plants for Human Health Institute, North Carolina State University, Kannapolis, NCUnited States; Department of Horticulture, North Carolina State University, Kannapolis, NCUnited States; Department of Horticulture, Michigan State University, East Lansing, MI, 48824, United States; Genetics and Genome Sciences, Michigan State University, East Lansing, MI, 48824, United States; MSU AgBioResearch, Michigan State University, East Lansing, MI, 48824, United States

## Abstract

Domestication of cranberry and blueberry began in the United States in the early 1800s and 1900s, respectively, and in part owing to their flavors and health-promoting benefits are now cultivated and consumed worldwide. The industry continues to face a wide variety of production challenges (e.g. disease pressures), as well as a demand for higher-yielding cultivars with improved fruit quality characteristics. Unfortunately, molecular tools to help guide breeding efforts for these species have been relatively limited compared with those for other high-value crops. Here, we describe the construction and analysis of the first pangenome for both blueberry and cranberry. Our analysis of these pangenomes revealed both crops exhibit great genetic diversity, including the presence–absence variation of 48.4% genes in highbush blueberry and 47.0% genes in cranberry. Auxiliary genes, those not shared by all cultivars, are significantly enriched with molecular functions associated with disease resistance and the biosynthesis of specialized metabolites, including compounds previously associated with improving fruit quality traits. The discovery of thousands of genes, not present in the previous reference genomes for blueberry and cranberry, will serve as the basis of future research and as potential targets for future breeding efforts. The pangenome, as a multiple-sequence alignment, as well as individual annotated genomes, are publicly available for analysis on the Genome Database for Vaccinium—a curated and integrated web-based relational database. Lastly, the core-gene predictions from the pangenomes will serve useful to develop a community genotyping platform to guide future molecular breeding efforts across the family.

## Introduction

The heath family (Ericaceae) contains many culturally and economically important berry crops, including bilberry (*Vaccinium myrtillus* L.), blueberry (*Vaccinium* spp. L.), cranberry (*Vaccinium macrocarpon* Aiton), huckleberry (*Vaccinium membranaceum* Douglas ex Torr.), and lingonberry (*Vaccinium vitis-idaea* L.) [1]. The common name ‘blueberry’ is applied to multiple *Vaccinium* species, including highbush blueberry (*Vaccinium corymbosum* L.), lowbush blueberry (*Vaccinium angustifolium* Aiton), and rabbiteye blueberry (*V. virgatum* Aiton [synonym = *V. ashei* J.M. Reade]) [2, 3]. Worldwide demand and consumption of cranberries and blueberries has rapidly increased over the past decades in large part owing to their health-promoting properties [4, 5]. Both cranberry and highbush blueberry are native to North America [6, 7]. Cranberry is a diploid species (2n = 2x = 24), whereas highbush blueberry is a tetraploid (2n = 4x = 48) [8, 9]. Highbush blueberry is further subdivided into northern and southern varieties that were selected largely for their overall chilling requirements for flowering and winter hardiness differences [10]. The cultivation of cranberry and highbush blueberry began in the early 1800s and 1900s, respectively [11, 12]. Thus, the domestication history of these species is much shorter than those of other crops, and there remains a great unexplored breeding potential for all *Vaccinium* crops [13].

Recent studies have shown that sequencing a single reference genotype or cultivar of a species is insufficient to recover all the genetic diversity present in a group [14–17]. This was first recognized in microbial studies; sets of genes were found to either be present in every member of a population (core) or absent in at least a single individual (dispensable) [18]. Here, we choose to refer to dispensable genes as auxiliary genes [19]. Although absent in some individuals, certain auxiliary genes if lost in combination can be lethal because of either redundancy or epistatic interactions with other auxiliary genes [20]. The sum of all core and auxiliary genes is termed a pangenome [21]. Several pangenome studies have been conducted in plants including *Brachypodium distachyon*, *Brassica napus*, maize, soybean, rice, and strawberry [22–27].

Core genes are consistently found to be enriched for ‘housekeeping’ functions including essential metabolic processes, whereas auxiliary genes were found to be enriched for more adaptive functions. For example, in *Brassica oleracea* (European cabbage), auxiliary genes are strongly enriched for defense response and specialized metabolism, including those that contribute to unique flavor profiles and vitamin content [28]. In *B. distachyon*, auxiliary genes were similarly enriched for defense functions, as well as other adaptive traits that are of particular interest for breeding superior crop varieties [22]. In addition, auxiliary genes often display signatures of elevated sequence turnover and relaxed selection and are shorter relative to core genes [29].

The proportion of core and auxiliary genes identified can vary for a given species or crop type. This value ranges from 33% to 80% of core genes. For example, about 80% of genes in rice (*Oryza sativa*) are core, while in corn (*Zea mays*), roughly 40% of genes are core [27, 30]. For a summary of core gene predictions across several plant pangenome studies, see Golicz *et al.* [17]. Life history characteristics and representative divergence likely contribute to the relative amount of auxiliary genes present for a particular species pangenome [31]. Furthermore, the rates of structural variation also contribute to pangenome size characteristics [32].

Characterization of auxiliary genes is crucial to maximize the impacts of molecular breeding approaches [33]. Genes underlying many important target traits (e.g. metabolites associated with fruit quality or disease resistance) are often auxiliary [24]. Previous genome-wide association studies (GWAS) have uncovered several additional candidate loci controlling traits of interest when leveraging a pangenome [31, 34]. For example, one GWAS in pigeon pea uncovered a gene associated with seed weight that was absent in the primary reference genome [35]. Similarly, Song et al. (2020) leveraged presence–absence variation information from eight reference quality *Brassica* genomes to perform a GWAS and identified novel transposable element insertions associated with variation in flowering time and silique weight [36]. This illustrates the translational impact of extending analyses beyond single reference genome frameworks.

Here, we generated and annotated genomes for 10 diverse cranberry and 20 diverse highbush blueberry cultivars. In conjunction with a previously published reference genome for these crops [37–39], we developed a pangenome for these crop species separately, and combined for both species and estimated the core genome size across the genus *Vaccinium.* In addition, we explored distinguishing features between core and auxiliary genes in blueberry and cranberry. These genomic resources and our pangenome estimates will serve as a powerful resource to guide future molecular breeding efforts and genetic studies across the *Vaccinium* community.

## Results

### Selection of accessions, sequencing, assembly, and annotation

We selected 10 cranberry cultivars, 10 southern highbush blueberry cultivars, and 10 northern highbush blueberry cultivars for genome sequencing and annotation. For cranberry and blueberry, reference genomes for cultivars ‘Stevens’ and ‘Draper’, respectively, were published previously and included in our analyses [37, 38]. Cultivars were selected based on genetic marker and pedigree analysis to capture the greatest amount of diversity [40] (Figure S1). Accessions were sequenced to an average depth of 112.5X for cranberry and 53.8X for blueberry across each haplotype or 225X for cranberry and 215.2X for blueberry in comparison to a single haplotype reference genome (Table S1). A hybrid reference-based and *de novo* assembly method was used to assemble genomes for each individual [41]. To guide annotation, RNA-seq data were collected from leaf and berry tissue. Each of the genomes were annotated using MAKER2, using the aforementioned RNAseq data, producing on average 27 856 and 105 523 genes for cranberry and blueberry, respectively. Our genome assembly qualities closely reflect those of the ‘Stevens’ (cranberry), ‘Draper’ (northern highbush blueberry), and ‘Arcadia’ (southern highbush blueberry) reference genomes [37, 38]. ‘Draper’ and ‘Arcadia’ are haplotype phased reference genomes, which is thus why roughly four times as many genomes were identified for blueberry than for cranberry. Scaffold N50 values were ~ 37 Mb for northern highbush genomes, ~1.4 Mb for southern highbush genomes, and ~ 38 Mb for cranberry genomes (Table S1). Complete BUSCO scores ranged from 82.9% to 91.7% (Table S1).

### Identification of cranberry core and auxiliary genes

**Figure 1:**
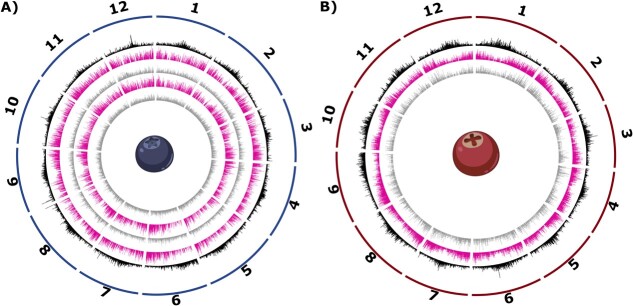
Circos plots for the blueberry and cranberry pangenomes. Circos plots for the blueberry (panel A) and cranberry (panel B) pangenomes are shown. Tracks display feature density of 50 kilobase windows. Tracks from exterior to interior are as follows: (1) karyotype of the 12 main pseudomolecules, (2) density of LTR transposons in black (second outer-most track), (3) density of core genes in pink (third and fifth track in panel A,third track in panel B), and (4) density of auxiliary genes in gray (fourth and innermost track in panel A, innermost track in panel B). For panel A, northern highbush blueberry is shown on the third and fourth tracks, while the two innermost tracks reflect southern highbush blueberry core and auxiliary genes.

All eleven cranberry genomes were aligned using ProgressiveCactus [42]. To identify core and auxiliary genes in cranberry, we integrated results from Orthofinder2 and our genome alignment [43]. For core gene classification, we search for genes present in each individual. Orthofinder2 alone will identify the presence of gene family members. Therefore, to integrate syntenic information, we filtered for the presence of alignments in our ProgressiveCactus results. Since annotations and genomes were generated for all eleven cranberry genotypes, we could label every gene (302 090 total) as either core or auxiliary. Of the roughly 27 463 genes, on average per accession, 14 553 (53%) and 12 910 (47%) genes were identified as core and auxiliary respectively (Figure 1).

### Identification of blueberry core and auxiliary genes

Highbush blueberry is a tetraploid and the ‘Draper’ genome assembly for highbush blueberry consists of four haplotypes [37]. The origin of highbush blueberry was previously estimated to either be an allopolyploid formed by the interspecific hybridization of two closely related species or an autopolyploid derived from the hybridization of two highly divergent populations of a single ancestral species [37]. Based on the relatively high sequence divergence at synonymous sites between the four homoeologs, it was concluded that the origin of tetraploid blueberry is unlikely an autopolyploid that was formed involving a single individual (parent) [37]. The most recent common ancestor of the diploid progenitors of highbush blueberry was estimated between 0.94 and 1.02 million years ago [37]. However, chromosomes do exhibit multivalent pairing (i.e. autopolyploid-like inheritance) in highbush blueberry [39]. It’s important to note that an allopolyploid that behaves like an autopolyploid during meiosis isn’t unique to highbush blueberry. For example, *B. napus* (rapeseed), is an allopolyploid formed by the interspecific hybridization of *B. oleracea* (European cabbage) and *Brassica rapa* (Chinese cabbage), each species with a distinct base chromosome number (n = 9 and n = 10), but some of those chromosomes still exhibit autopolyploid-like multivalent pairing during meiosis.

**Figure 2:**
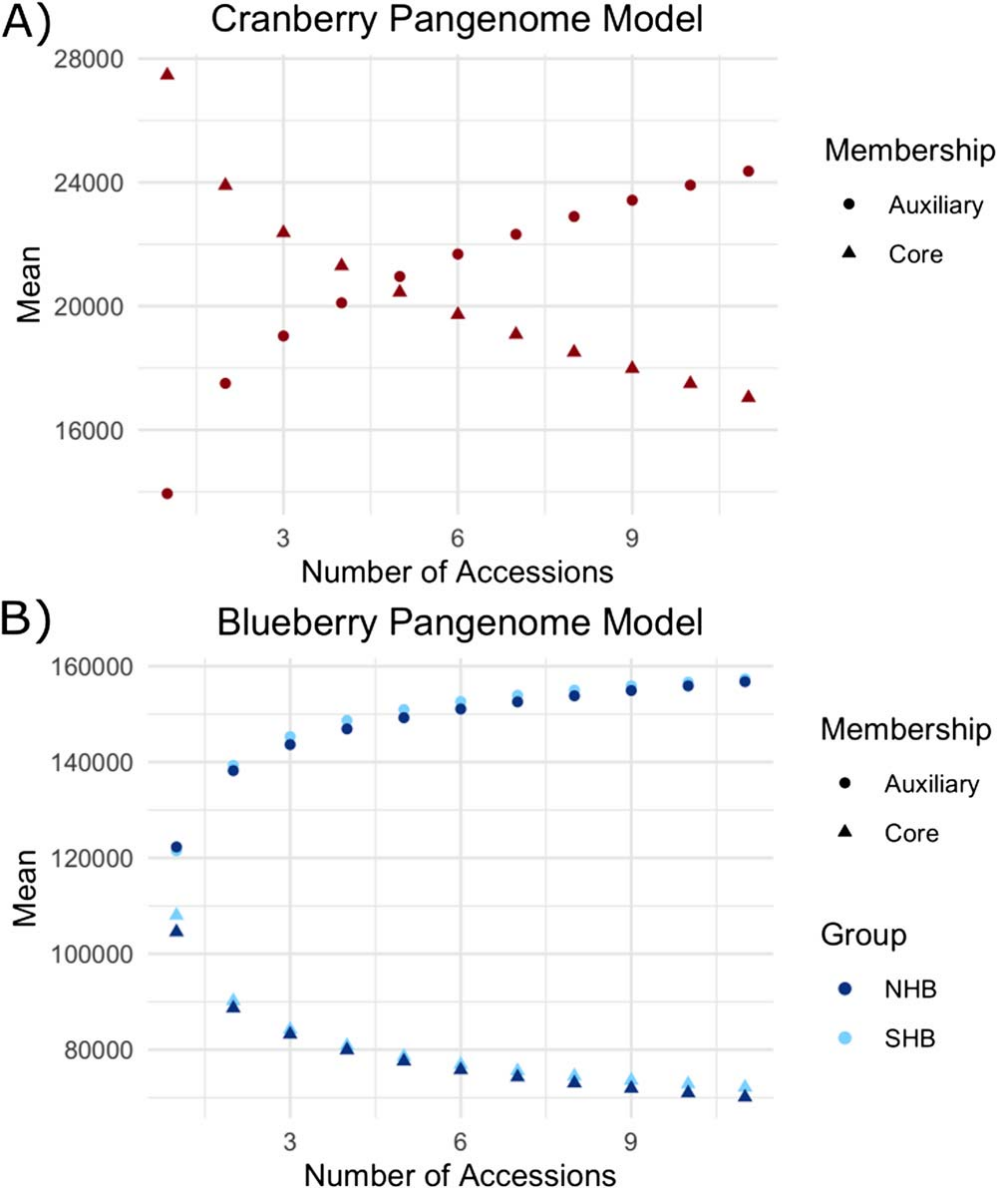
Core and auxiliary genome modeling for cranberry and blueberry. Panel A depicts the model for the core (circle) and auxiliary (triangle) pangenome for cranberry. For each point along the x-axis, we take every possible combination of that size from our genome samples and plot the average number of core and auxiliary genes as a point. Panel B depicts the model for the core (triangles) and auxiliary genes (circles) for northern (dark blue) and southern (light blue) highbush blueberry. Note the y-axes do not start at zero.

Due to the polyploidy, calling core and auxiliary genes is complicated by the differential presence or absence of genes on different haplotypes. Gene fractionation (loss) in polyploid genomes is expected and will inflate auxiliary designations [44]. To facilitate core and auxiliary gene designations, we compared our blueberry assemblies with a recently published diploid genotype (W85) for blueberry [39] to accurately assess the presence or absence of each gene in the northern and southern highbush blueberry genome assemblies. The W85 genome assembly consists of phased haplotypes with 34 848 gene models in the primary haplotype (p0) and 33 148 in the alternate haplotype (p1). As pangenome patterns were largely consistent between haplotypes, the rest of our analyses focused on the primary ‘p0’ haplotype. Of these, 45.80% (n = 15 150) were present in all highbush blueberry cultivars based on purely genome synteny analyses. Northern highbush blueberry exhibited a higher proportion of core genes (53.87%) than southern highbush blueberry (48.78%), and similar numbers were estimated for each haplotype. We also analyzed core genome size using OrthoFinder2, as we did above with cranberry, and identified 14 956 (51.6%) as core orthogroups and 14 042 (48.4%) auxiliary orthogroups shared across all highbush blueberry cultivars (Figure 2). Orthogrouping permitted us to identify core genes that were no longer in the ancestral position but still present in the genome.

### Pangenome modeling

Though cultivars were selected to capture the greatest genetic diversity (Figure S1), to capture all genetic diversity we must assay every individual. However, we can estimate the size of the pangenome through modeling our sample. Figure 2 displays this model of the core and auxiliary gene content of the pangenome estimated using orthogrouping to best capture core and auxiliary genes that were no longer in their ancestral position in the genome. For cranberry, we identified 14 552 core genes out of an average of 27 462 total genes per accession (53%). The number of total auxiliary genes increases as more genomes are queried. However, the amount of new auxiliary genes per accession added decreases. The pangenome is considered ‘closed’ when the total number of auxiliary genes eventually reaches a plateau. That plateau has not been reached modeling on our data. Therefore, further sampling of cranberry genomes will uncover greater genetic variation and additional novel auxiliary genes.

For blueberry, the model of auxiliary and core gene content appeared to reach a plateau more quickly than cranberry. This suggests the same numbers of blueberry individuals for both northern and southern highbush varieties captures a greater proportion of total auxiliary genes than for cranberry, and that we are closer to estimating the true core genome size for blueberry. However, we still observe an incomplete plateau and expect to uncover novel auxiliary genes and an improved core genome size as more genomes are queried. In northern highbush blueberry, there were an average of 70 073 core genes out of 104 552 total genes per accession (67.02%). In southern highbush blueberry, there were an average of 72 155 core genes out of an average of 108 020 total genes per accession (66.80%).

### Differences between auxiliary and core genes

**Figure 3:**
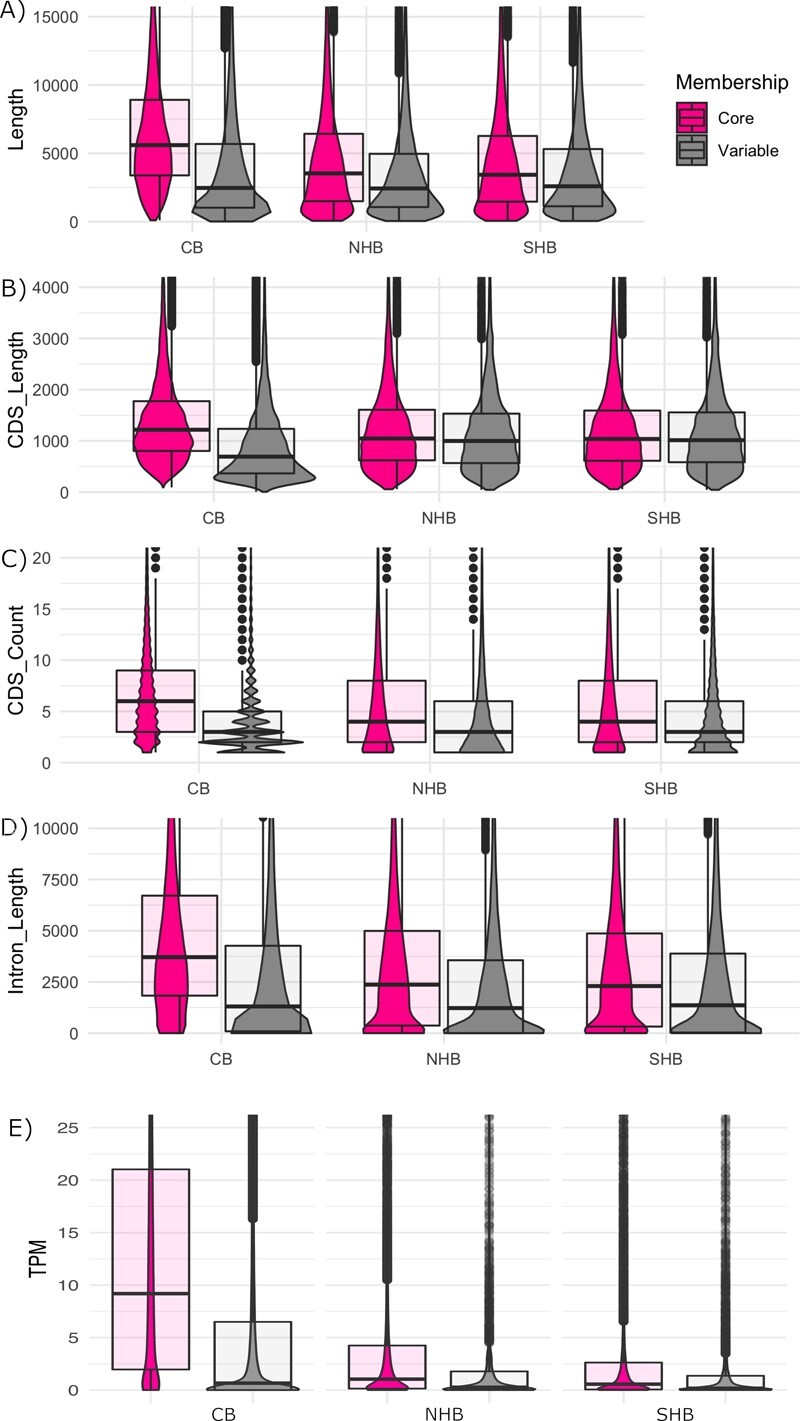
Differences between core and auxiliary genes. Density plots showing the differences between core (pink, left plot for each comparison) and auxiliary (gray, right plot for each comparison) genes for cranberry (CB), northern highbush blueberry (NHB) and southern highbush blueberry (SHB). We compared (A) gene length (transcription start site to transcription end site), (B) coding sequence length (CDS) length, (C) CDS count, (D) intron length, and (E) fruit transcripts per million (TPM).

Previous studies identified differences between core and auxiliary genes including gene length, exon count, and guanine-cytosine (GC) percentage [29, 45]. Here, we uncovered similar differences between these two groups of genes in both cranberry and blueberry. Figure 3 displays differences between core and auxiliary genes across cranberry (CB), northern highbush blueberry (NHB) and southern highbush blueberry (SHB). Auxiliary genes are dramatically shorter, and have both fewer and shorter introns than core genes. Furthermore, we found that the expression of core genes was higher on average than that of auxiliary genes (Figure 3).

Next, we compared functional differences between core and auxiliary genes (Table S2). For auxiliary genes in cranberry, we did observe a few expected enriched GO terms that were reported in previous studies [41] with the top three GO terms being ‘GO:0009607 response to biotic stimulus’ (FDR *P* = 1.22E-08), ‘GO:0043207 response to external biotic stimulus’ (FDR *P* = 1.66E-08), and ‘GO:0051707 response to other organism’ (FDR *P* = 1.66E-08). In addition, auxiliary genes were enriched with many other GO terms of significance to important target breeding traits, including ‘GO:0009631 cold acclimation’ (FDR *P* = 0.0353), ‘GO:0002213 defense response to insect’ (FDR *P* = 0.0041), and ‘GO:0050832 defense response to fungus’ (FDR *P* = 0.0031). Furthermore, we observed enrichment for several GO terms relevant to flavonoids, a group of specialized metabolites, previously shown to affect the pigmentation, health benefits (antioxidant), and defense against pathogens in berries [46] (e.g. ‘GO:0009812 flavonoid metabolic process’ (FDR *P* = 0.0388), ‘GO:0051555 flavonol biosynthetic process’ (FDR *P* = 0.0023)). For cranberry, core genes were enriched for core biological processes, with the top three enriched GO terms being ‘GO:0019222 regulation of metabolic process’ (FDR *P* = 3.37E-08), ‘GO:00104 regulation of gene expression’ (FDR *P* = 8.36E-07), and ‘GO:0065007 biological regulation’ (FDR *P* = 1.76E-06).

Similarly, in blueberry, the top three enriched GO terms for auxiliary genes were ‘GO:0006952 defense response’ (FDR *P* = 6.10E-17), ‘GO:0009607 response to biotic stimulus’ (FDR *P* = 1.03E-14), and ‘GO:0044419 biological process involved in interspecies interaction between organisms’ (FDR *P* = 1.04E-14). In addition, auxiliary genes were enriched with many other GO terms of significance to important target breeding traits, including ‘GO:0071497 cellular response to freezing’ (FDR *P* = 0.0279), ‘GO:0050832 defense response to fungus’ (FDR *P* = 6.61E-07), and ‘GO:0009617 response to bacterium’ (FDR *P* = 4.49E-12). Furthermore, several GO terms relevant to the pigmentation, health benefits, aroma and flavor of berries are enriched among auxiliary genes (e.g. ‘GO:0009813 flavonoid biosynthetic process’ (FDR *P* = 0.0009), ‘GO:0019745 pentacyclic triterpenoid biosynthetic process’ (FDR *P* = 0.0249), and ‘GO:0042335 cuticle development’ (FDR *P* = 0.0037)). For core genes in blueberry, the top three enriched GO terms were ‘GO:0006396 RNA Processing’ (FDR *P* = 3.76E-31), ‘GO:0034641 cellular nitrogen compound metabolic process’ (FDR *P* = 1.26E-21), and ‘GO:0034660 ncRNA metabolic process’ (FDR *P* = 4.78E-21).

### Individual genomes and pangenome resources available on public database

**Figure 4:**
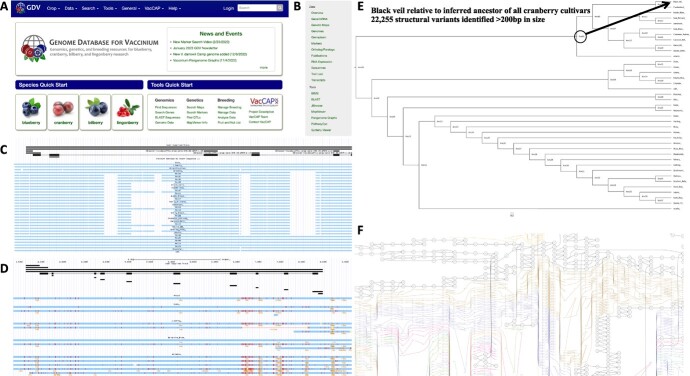
Pangenomic resources available on Genome Database for *Vaccinium.* All genomic resources developed here are publicly available for the community to use on the Genome Database for Vaccinium (GDV; https://www.vaccinium.org/) (Panel A). A wide variety of tools are available to analyze individual genomes and/or compare genomes (Panel B). A multiple sequence alignment is available to identify structural variants, including deletions (Panel C), single nucleotide polymorphisms (Panel D), and place genetic variants into a phylogenetic context (Panel E). Panel F depicts a pangenome graph view of the multiple sequence alignment.

Each of the assembled highbush blueberry and cranberry genomes, alongside gene and repeat annotations, are now publicly available on the Genome Database for Vaccinium [47] (Figure 4A). The Genome Database for Vaccinium (GDV) is a curated and integrated web-based relational database to house and integrate genomic, genetic and breeding data for *Vaccinium* species. Members of the community can visit GDV to view a gene(s) of any genome in JBrowse, search for one or more sequences using BLAST, use synteny viewer to display all the conserved syntenic blocks between any genomes, and pathway predictions including for those that encode specialized metabolites associated with superior fruit quality (Figure 4B). For example, presence-absence variation of genes involved in the biosynthesis of flavonoids was recently assessed across the blueberry pangenome [48]. In addition, the multiple whole genome alignment is available that can be used to view structural variants, including insertion/deletions (InDels) (Figure 4C), and small polymorphisms (Figure 4D). ProgressiveCactus [42] was also used to estimate ancestral states along a phylogeny of the sequenced genome, which can be leveraged to determine the number of events that occurred along a particular branch. For example, we identified 22 255 structural variants >200 bp that occurred since the most common recent ancestor of all eleven cranberry and present in ‘Black Veil’ (Figure 4E). A complete list of all identified variants, ranging from small single nucleotide substitutions to larger structural events (insertions, deletions, inversions, duplications, and transpositions) are available in Table S3. In addition, pangenome variation graphs for blueberry and cranberry loci can be generated from the hierarchical alignment (HAL) on the GDV (see for example Figure 4F).

**Figure 5:**
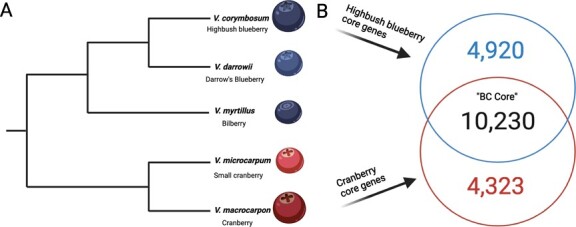
Conservation of highbush blueberry-cranberry (BC) core gene content. Panel A depicts the phylogenetic relationships among five *Vaccinium* species, previously estimated by [49]. Panel B depicts the comparison of the highbush blueberry and cranberry syntenically conserved core genes.

### Pangenome conservation and as a new resource for the development of future breeding tools

Blueberry and cranberry species belong to two distinct and distantly related clades that diverged between 5 and 10 million years ago (Figure 5A) [49]. We evaluated the conservation of positionally conserved core genes between highbush blueberry and cranberry, which revealed a Blueberry-Cranberry ‘BC Core’ consisting of 10 230 genes (Figure 5B). A total of 4920 and 4323 core genes were identified as highbush blueberry and cranberry specific, respectively. Next, we evaluated the conservation of the ‘BC Core’ gene content in comparison to other *Vaccinium* species with available genomes. In the small cranberry (*Vaccinium microcarpum*) genome [38], roughly 95.5% (9767 total) genes from the ‘BC Core’ were identified and positionally conserved in the genome. For bilberry (*V. myrtillus*) [50] and Darrow’s blueberry (*Vaccinium darrowii*) [51], roughly 94.5% (9672 total) and 91.3% (9345 total) genes, respectively, from the ‘BC Core’ were identified and positionally conserved in the genomes. A major goal of the VacCAP project [52], a multi-institutional and multi-disciplinary project, is the assembly of a pangenome in order to construct a robust genotyping platform that can be used for molecular breeding efforts for blueberry, cranberry and potentially other related cultivated *Vaccinium* species. Here, we identified a set of 10 230 positionally conserved core genes that span each chromosome of both blueberry and cranberry species, and are largely present (>90%) in related *Vaccinium* species in positionally conserved regions of the genome.

## Discussion

In this study, we defined genes in highbush blueberry and cranberry as core or auxiliary based on their presence or absence across diverse cultivars. In cranberry, we uncovered 53% of all genes being core and 47% as auxiliary genes. In blueberry, we uncovered similar rates, with 51% of all genes being core and 49% as auxiliary genes. The proportions of core and auxiliary genes identified for blueberry and cranberry were similar to those of other crops and species, including maize [27], *Brachypodium* [22] and strawberry [26]. However, not all crop species exhibit presence-absence variation that impacts roughly half of the gene content. Three main factors affect the proportion of core genes that may be identified for a species: divergence time of the genotypes compared, extant diversity of the species and life history traits.

Previous studies have also identified characteristic differences between core and auxiliary genes [29]. These differences lend insight into core/auxiliary gene function as well as their origin and subsequent evolution. Both core and auxiliary gene characteristic distributions overlap. Therefore, dichotomies cannot be drawn between these two classes. Rather, as with most biological processes, they exist on a continuum. That being said, our identified characteristic differences tell us auxiliary genes show patterns of evolutionarily young genes and are enriched with more adaptive functions that are important breeding targets.

Shorter sequences for novel genes support two models for gene birth: (1) *de novo* emergence [53] and (2) duplication degeneration [54, 55]. Determining a specific mechanism for gene birth for these auxiliary genes is beyond the scope of this work. However, reports of *de novo* gene origin in yeast and *Drosophila* show evidence of novel genes arising from previously short noncoding DNA sequences [56, 57]. Furthermore, small-scale duplications present an abundance of substrate for evolution to shape novel genes [58]. One mechanism through which gene duplication leads to novel gene function is through neofunctionalization and/or subfunctionalization [59, 60]. After a gene duplicates, one copy can explore the evolutionary landscape without detrimental selective impacts because the other copy can compensate for loss or change of function. This can lead to fractionation of either or both copies and possibly reflect the shorter distribution of auxiliary genes.

We found that core and auxiliary enriched gene functions in *Vaccinium* align with observations in previous pangenome studies. Auxiliary genes are enriched for adaptive functions (e.g. defense response) similar to those of *B. distachyon* [22] while core genes are enriched for basic cellular processes similar to those in *B. oleracea* [28]. Auxiliary genes are therefore targets of phenotypic differences between individuals and are of high agronomic importance. We discovered a large proportion of auxiliary genes in blueberry and cranberry that range between cultivar- to clade- to species-specific. This represents a substantial gene pool to leverage for future breeding efforts. Furthermore, genome-wide association studies (GWAS) can leverage pangenomes to discover genetic variants associated with important target traits [34, 36]. This may be a critical next step to further dissect the underlying genetics encoding important traits for *Vaccinium* breeding [13].

Lastly, the positionally-conserved core genes identified in this study can serve as foundational markers for the development of a genotyping platform for future molecular breeding efforts that is effective across a diversity of genetic backgrounds. In addition, certain auxiliary and/or species-specific genes, including those that contribute to improved disease resistance, stress tolerances and fruit quality traits, may serve useful to include on a genotyping platform for the blueberry and cranberry breeding community. Future research efforts will be focused on further characterizing these auxiliary genes and identifying those that greatly impact important target traits in both blueberry and cranberry.

## Materials and methods

### Genome sequencing, assembly, and annotation

For genomic sequencing, leaf tissue was collected from each of the cultivars selected to best represent the genetic diversity of cranberry, southern highbush blueberry and northern highbush blueberry. DNA was extracted using a DNeasy extraction kit (Qiagen, Hilden, Germany). DNA quantity was assessed using a Qubit (ThermoFisher, Waltham, Massachusetts). Genomic libraries were prepared with the HyperPrep Library construction kit from Kapa Biosystems (Roche, Basel, Switzerland). Libraries were sequenced on a NovaSeq 6000 (Illumina, San Diego, CA) in the Michigan State University Research Technology Support Facility (MSU RTSF) using a NovaSeq S4 reagent kit for 151 cycles from each end to generate paired 150 nucleotide long reads.

Genomic reads were quality and adapter trimmed using trimmomatic version 0.38 [61]. Reads were then used to generate a hybrid *de novo* and reference based genome assembly. This assembly method was described in detail previously including tool versions and command line options [62]. Briefly, genomic reads were mapped to the reference genome generated previously for cranberry [38], northern highbush blueberry [37], and southern highbush blueberry (‘Arcadia’, early access provided by Patricio Muñoz). Mapped reads were used to generate a consensus genome sequence iteratively for three rounds. Then, unmapped reads were collected and *de novo* assembled into synthetic long-reads. These long reads were combined with the consensus sequence and incorporated into the final genome assembly for each cultivar.

Several tissues were collected for RNA sequencing analysis for each blueberry and cranberry accession, including young leaf, mature leaf, green berry, and mature berry. Total RNA was isolated using the RNAeasy extraction kit (Qiagen, Hilden, Germany). RNA quantity was assessed using a Qubit (ThermoFisher, Waltham, MA). RNA libraries were prepared from a pool of the aforementioned tissues according to the mRNA HyperPrep kit protocol (Roche, Basel, Switzerland). All samples were sequenced in the MSU RTSF Genomics core with paired-end 150 bp reads on an HiSeq 6000 system (Illumina). Reads were quality and adapter trimmed using trimmomatic version 0.38 [61]. They were mapped to their respective genome assemblies using hisat2 version 2.1.0 [63]. The resulting SAM files were sorted and converted to BAM files using PicardTools version 2.18.1 SortSam function. From these alignments, transcriptome assemblies were generated using Stringtie version 2.1.3 [64]. These transcriptome assemblies were used later to guide the gene annotation.

Each genome was annotated for protein coding genes using the MAKER2 pipeline [65]. Briefly, the genomes were masked for repetitive sequences via RepeatModeler. Proteins from Araport11 [66] and transcriptomes generated above were used as evidence. We also included the *V. corymbosum* ‘Draper’ CDS predictions [37] as evidence. We generated two *ab initio* models trained on ‘Draper’ gene models, SNAP and Augustus. Augustus models were generated using the script `train_augustus_draper.sh` on a subset of 4000 randomly selected gene models. SNAP models were generated using the script `train_snap_draper.sh`.

### Transposable element annotation

EDTA v2.0.0 was used to generate a pan-genome TE annotation [67–75]. Default parameters were used in all cases except for the usage of the `—sensitive 1` parameter which employs RepeatModeler to identify remaining TEs. First, individual repeat libraries were generated independently for each genome. Then, these libraries were filtered and combined using EDTA’s `make_panTElib.pl` script to generate a pangenome repeat library for each genome group. Finally, the pangenome repeat library was used to re-annotate each source genome. Scripts and documentation for this analysis can be found at: https://github.com/sjteresi/Vaccinium_Pangenome_TE_Analysis.

### Identification of core and auxiliary genes

We aligned each genome using progressiveCactus to obtain whole genome alignments to identify positionally conserved core genes and identify missing auxiliary genes in cranberry [42, 43]. The progressiveCactus alignment also includes the highbush blueberry genomes. For blueberry, SynMap within CoGe using LAST and default parameters against the diploid W85 blueberry genome was used to identify positionally conserved core genes and auxiliary genes [76]. We then identified orthologs between all cranberry and all blueberry proteomes using Orthofinder2 version 2.4.1 using default parameters (only the working directory specified) [43]. As Orthofinder2 might identify members of the same gene family as orthologous, we decided to filter out any ortholog calls without an alignment within 5 kbp of each other. We used the `filter_orthofinder2.sh` script for ortholog calls.

### Gene statistic calculations

We calculated several feature values for each gene model including: gene length, coding sequence length, exon count, intron count, exon length, and intron length. These values were calculated using the `annotate_core_genes_vacc_pan.py` script. Expression values were calculated using Kallisto v0.46.1 [77].

### Functional enrichments

Each proteome was functionally annotated using InterPro Scan version 5.28–67.0 [78]. We converted the InterPro Scan annotation ID to a gene ontology ID using a manually curated translation table. We performed gene ontology term enrichment difference between core and auxiliary genes in R using the script `vacc_pan_go_enrichment.Rmd` with the topGO package.

### Pangenome modeling

We modeled the core and auxiliary genomes of both blueberry and cranberry pangenomes based on the orthofinder results. We parsed the `Orthogroups.csv` and `Orthogroups_UnassignedGenes.csv` files using a custom script `model_pangenome_orthofinder.py`. This script calculates the number of core and auxiliary genes for each combination of individuals from 1 to the number of total accessions. We then plotted the average of core and auxiliary genes for each possible combination of accessions in Figure 2.

## Supplementary Material

VaccPan_supplementary_tables_uhad202

halSummarizeMutations_uhad202

## References

[1] Stevens PF . A classification of the Ericaceae: subfamilies and tribes. Bot J Linn Soc. 1971;64:1–53

[2] Lyrene PM , Vorsa N, Ballington JR. Polyploidy and sexual polyploidization in the genus Vaccinium. Euphytica. 2003;133:27–36

[3] Ehlenfeldt MK , Polashock JJ, Ballington JR. *Vaccinium corymbodendron* Dunal as a bridge between taxonomic sections and ploidies in vaccinium: a work in progress. North American Blueberry Research and Extension Workers Conference. 2018;15:1–7

[4] Silva S , Costa EM, Veiga M. et al. Health promoting properties of blueberries: a review. Crit Rev Food Sci Nutr. 2020;60:181–200 30373383 10.1080/10408398.2018.1518895

[5] Skrovankova S , Sumczynski D, Mlcek J. et al. Bioactive compounds and antioxidant activity in different types of berries. Int J Mol Sci. 2015;16:24673–706 26501271 10.3390/ijms161024673 PMC4632771

[6] Hancock JF , Draper AD. Blueberry culture in North America. HortScience. 1989;24:551–6

[7] Vorsa N , Zalapa J. Domestication, genetics, and genomics of the American cranberry. Plant Breeding Reviews. 2019;43:279–315

[8] Eck P . Response of the American cranberry to phosphorus fertilizer. Acta Hortic. 1985;165:299–302

[9] Coville FV . Blueberry chromosomes. Science. 1927;66:565–6 17820511 10.1126/science.66.1719.565

[10] Gough RE . The Highbush Blueberry and its Management. Boca Raton: CRC Press, 1993

[11] Ehlenfeldt MK . Domestication of the highbush blueberry at Whitesbog, New Jersey, 1911--1916. Acta Hortic. 2009;810:147–152

[12] Vorsa N , Johnson-Cicalese J. American Cranberry. In: Badenes ML, Byrne DH, eds. Fruit Breeding. Springer US: Boston, MA, 2012,191–223

[13] Edger PP , Iorizzo M, Bassil NV. et al. There and back again; historical perspective and future directions for Vaccinium breeding and research studies. Hortic Res. 2022;9:uhac083 35611183 10.1093/hr/uhac083 PMC9123236

[14] Qin P , Lu H, Du H. et al. Pan-genome analysis of 33 genetically diverse rice accessions reveals hidden genomic variations. Cell. 2021;184:3542–3558.e16 34051138 10.1016/j.cell.2021.04.046

[15] Varshney RK , Roorkiwal M, Sun S. et al. A chickpea genetic variation map based on the sequencing of 3,366 genomes. Nature. 2021;599:622–7 34759320 10.1038/s41586-021-04066-1 PMC8612933

[16] Hufford MB , Seetharam AS, Woodhouse MR. et al. De novo assembly, annotation, and comparative analysis of 26 diverse maize genomes. Science. 2021;373:655–62 34353948 10.1126/science.abg5289 PMC8733867

[17] Golicz AA , Bayer PE, Bhalla PL. et al. Pangenomics comes of age: from bacteria to plant and animal applications. Trends Genet. 2020;36:132–45 31882191 10.1016/j.tig.2019.11.006

[18] Medini D , Donati C, Tettelin H. et al. The microbial pan-genome. Curr Opin Genet Dev. 2005;15:589–94 16185861 10.1016/j.gde.2005.09.006

[19] Breitbart M , Thompson LR, Suttle CA. et al. Exploring the vast diversity of marine viruses. Oceanography. 2007;20:135–9

[20] Marroni F , Pinosio S, Morgante M. Structural variation and genome complexity: is dispensable really dispensable? Curr Opin Plant Biol. 2014;18:31–6 24548794 10.1016/j.pbi.2014.01.003

[21] Tettelin H , Masignani V, Cieslewicz MJ. et al. Genome analysis of multiple pathogenic isolates of *Streptococcus agalactiae*: implications for the microbial ‘pan-genome’. Proc Natl Acad Sci U S A. 2005;102:13950–5 16172379 10.1073/pnas.0506758102 PMC1216834

[22] Gordon SP , Contreras-Moreira B, Woods DP. et al. Extensive gene content variation in the *Brachypodium distachyon* pan-genome correlates with population structure. Nat Commun. 2017;8:2184 29259172 10.1038/s41467-017-02292-8 PMC5736591

[23] Hurgobin B , Golicz AA, Bayer PE. et al. Homoeologous exchange is a major cause of gene presence/absence variation in the amphidiploid *Brassica napus*. Plant Biotechnol J. 2018;16:1265–74 29205771 10.1111/pbi.12867 PMC5999312

[24] Li Y-H , Zhou G, Ma J. et al. De novo assembly of soybean wild relatives for pan-genome analysis of diversity and agronomic traits. Nat Biotechnol. 2014;32:1045–52 25218520 10.1038/nbt.2979

[25] Wang W , Mauleon R, Hu Z. et al. Genomic variation in 3,010 diverse accessions of Asian cultivated rice. Nature. 2018;557:43–9 29695866 10.1038/s41586-018-0063-9 PMC6784863

[26] Qiao Q , Edger PP, Xue L. et al. Evolutionary history and pan-genome dynamics of strawberry (spp.). Proc Natl Acad Sci U S A. 2021;118:118 10.1073/pnas.2105431118 PMC8609306 34697247

[27] Hirsch CN , Foerster JM, Johnson JM. et al. Insights into the maize pan-genome and pan-transcriptome. Plant Cell. 2014;26:121–35 24488960 10.1105/tpc.113.119982 PMC3963563

[28] Golicz AA , Bayer PE, Barker GC. et al. The pangenome of an agronomically important crop plant *Brassica oleracea*. Nat Commun. 2016;7:13390 27834372 10.1038/ncomms13390 PMC5114598

[29] Yocca AE , Edger PP. Machine learning approaches to identify core and dispensable genes in pangenomes. *Plant*. Genome. 2022;15:1–11 10.1002/tpg2.20135 34533282

[30] Zhao Q , Feng Q, Lu H. et al. Pan-genome analysis highlights the extent of genomic variation in cultivated and wild rice. Nat Genet. 2018;50:278–84 29335547 10.1038/s41588-018-0041-z

[31] Tao Y , Zhao X, Mace E. et al. Exploring and exploiting pan-genomics for crop improvement. Mol Plant. 2019;12:156–69 30594655 10.1016/j.molp.2018.12.016

[32] Lei L , Goltsman E, Goodstein D. et al. Plant pan-genomics comes of age. Annu Rev Plant Biol. 2021;72:411–35 33848428 10.1146/annurev-arplant-080720-105454

[33] Tay Fernandez CG , Nestor BJ, Danilevicz MF. et al. Pangenomes as a resource to accelerate breeding of under-utilised crop species. Int J Mol Sci. 2022;23:23 10.3390/ijms23052671 PMC8910360 35269811

[34] Zhou Z , Jiang Y, Wang Z. et al. Resequencing 302 wild and cultivated accessions identifies genes related to domestication and improvement in soybean. Nat Biotechnol. 2015;33:408–14 25643055 10.1038/nbt.3096

[35] Zhao J , Bayer PE, Ruperao P. et al. Trait associations in the pangenome of pigeon pea (*Cajanus cajan*). Plant Biotechnol J. 2020;18:1946–54 32020732 10.1111/pbi.13354 PMC7415775

[36] Song J-M , Guan Z, Hu J. et al. Eight high-quality genomes reveal pan-genome architecture and ecotype differentiation of *Brassica napus*. Nat Plants. 2020;6:34–45 31932676 10.1038/s41477-019-0577-7 PMC6965005

[37] Colle M , Leisner CP, Wai CM. et al. Haplotype-phased genome and evolution of phytonutrient pathways of tetraploid blueberry. GigaScience. 2019;8:8 10.1093/gigascience/giz012 PMC6423372 30715294

[38] Diaz-Garcia L , Garcia-Ortega LF, González-Rodríguez M. et al. Chromosome-level genome assembly of the American cranberry (*Vaccinium macrocarpon* Ait.) and its wild relative *Vaccinium microcarpum*. Front Plant Sci. 2021;12:1–12 10.3389/fpls.2021.633310 PMC7902871 33643360

[39] Mengist MF , Bostan H, De Paola D. et al. Autopolyploid inheritance and a heterozygous reciprocal translocation shape chromosome genetic behavior in tetraploid blueberry (*Vaccinium corymbosum*). New Phytol. 2023;237:1024–39 35962608 10.1111/nph.18428 PMC10087351

[40] Bassil N , Bidani A, Nyberg A. et al. Microsatellite markers confirm identity of blueberry (*Vaccinium* spp.) plants in the USDA-ARS National Clonal Germplasm Repository collection. Genet Resour Crop Evol. 2020;67:393–409

[41] Golicz AA , Bayer PE, Barker GC. et al. The pangenome of an agronomically important crop plant *Brassica oleracea*. Nature. Communications. 2016;7:7 10.1038/ncomms13390 PMC5114598 27834372

[42] Armstrong J , Hickey G, Diekhans M. et al. Progressive cactus is a multiple-genome aligner for the thousand-genome era. Nature. 2020;587:246–51 33177663 10.1038/s41586-020-2871-y PMC7673649

[43] Emms DM , Kelly S. OrthoFinder: phylogenetic orthology inference for comparative genomics. Genome Biol. 2019;20:238 31727128 10.1186/s13059-019-1832-y PMC6857279

[44] Freeling M , Scanlon MJ, Fowler JE. Fractionation and subfunctionalization following genome duplications: mechanisms that drive gene content and their consequences. Curr Opin Genet Dev. 2015;35:110–8 26657818 10.1016/j.gde.2015.11.002

[45] Danilevicz MF , Tay Fernandez CG, Marsh JI. et al. Plant pangenomics: approaches, applications and advancements. Curr Opin Plant Biol. 2020;54:18–25 31982844 10.1016/j.pbi.2019.12.005

[46] Panche AN , Diwan AD, Chandra SR. Flavonoids: an overview. J Nutr Sci. 2016;5:1–15 10.1017/jns.2016.41 PMC5465813 28620474

[47] GDV . https://www.vaccinium.org/ (accessed 26 May2023).

[48] Albert NW , Iorizzo M, Mengist MF. et al. Vaccinium as a comparative system for understanding of complex flavonoid accumulation profiles and regulation in fruit. Plant Physiol. 2023;192:1696–710 37129240 10.1093/plphys/kiad250 PMC10315322

[49] Zhidkin RR , Matveeva TV. Phylogeny problems of the genus *Vaccinium* L. and ways to solve them. Ecological genetics. 2022;20:151–64

[50] Wu C , Deng C, Hilario E. et al. A chromosome-scale assembly of the bilberry genome identifies a complex locus controlling berry anthocyanin composition. Mol Ecol Resour. 2022;22:345–60 34260155 10.1111/1755-0998.13467

[51] Yu J , Hulse-Kemp AM, Babiker E. et al. High-quality reference genome and annotation aids understanding of berry development for evergreen blueberry (*Vaccinium darrowii*). Hortic Res. 2021;8:228 34719668 10.1038/s41438-021-00641-9 PMC8558335

[52] Home . https://www.vacciniumcap.org/ (accessed 30 May2023).

[53] Van Oss SB , Carvunis A-R. De novo gene birth. PLoS Genet. 2019;15:1–23 10.1371/journal.pgen.1008160 PMC6542195 31120894

[54] Conant GC , Wolfe KH. Turning a hobby into a job: how duplicated genes find new functions. Nat Rev Genet. 2008;9:938–50 19015656 10.1038/nrg2482

[55] Force A , Lynch M, Pickett FB. et al. Preservation of duplicate genes by complementary, degenerative mutations. Genetics. 1999;151:1531–45 10101175 10.1093/genetics/151.4.1531 PMC1460548

[56] Carvunis A-R , Rolland T, Wapinski I. et al. Proto-genes and de novo gene birth. Nature. 2012;487:370–4 22722833 10.1038/nature11184 PMC3401362

[57] Siepel A . Darwinian alchemy: human genes from noncoding DNA. Genome Res. 2009;19:1693–5 19797681 10.1101/gr.098376.109 PMC2765273

[58] Ohno S . Evolution by gene duplication. 1970:1–160. 10.1007/978-3-642-86659-3

[59] Lynch M , Force A. The probability of duplicate gene preservation by subfunctionalization. Genetics. 2000;154:459–73 10629003 10.1093/genetics/154.1.459 PMC1460895

[60] Birchler JA , Yang H. The multiple fates of gene duplications: deletion, hypofunctionalization, subfunctionalization, neofunctionalization, dosage balance constraints, and neutral variation. Plant Cell. 2022;34:2466–74 35253876 10.1093/plcell/koac076 PMC9252495

[61] Bolger AM , Lohse M, Usadel B. Trimmomatic: a flexible trimmer for Illumina sequence data. Bioinformatics. 2014;30:2114–20 24695404 10.1093/bioinformatics/btu170 PMC4103590

[62] Yocca AE , Lu Z, Schmitz RJ. et al. Evolution of conserved noncoding sequences in *Arabidopsis thaliana*. Mol Biol Evol. 2021;38:2692–703 33565589 10.1093/molbev/msab042 PMC8233505

[63] Kim D , Paggi JM, Park C. et al. Graph-based genome alignment and genotyping with HISAT2 and HISAT-genotype. Nat Biotechnol. 2019;37:907–15 31375807 10.1038/s41587-019-0201-4 PMC7605509

[64] Shumate A , Wong B, Pertea G. et al. Improved transcriptome assembly using a hybrid of long and short reads with StringTie. PLoS Comput Biol. 2022;18:1–18 10.1371/journal.pcbi.1009730 PMC9191730 35648784

[65] Holt C , Yandell M. MAKER2: an annotation pipeline and genome-database management tool for second-generation genome projects. BMC Bioinformatics. 2011;12:491 22192575 10.1186/1471-2105-12-491 PMC3280279

[66] Cheng C-Y , Krishnakumar V, Chan AP. et al. Araport11: a complete reannotation of the *Arabidopsis thaliana* reference genome. Plant J. 2017;89:789–804 27862469 10.1111/tpj.13415

[67] Ou S , Su W, Liao Y. et al. Benchmarking transposable element annotation methods for creation of a streamlined, comprehensive pipeline. Genome Biol. 2019;20:275 31843001 10.1186/s13059-019-1905-y PMC6913007

[68] Ellinghaus D , Kurtz S, Willhoeft U. LTRharvest, an efficient and flexible software for de novo detection of LTR retrotransposons. BMC Bioinformatics. 2008;9:18 18194517 10.1186/1471-2105-9-18 PMC2253517

[69] Xu Z , Wang H. LTR_FINDER: an efficient tool for the prediction of full-length LTR retrotransposons. Nucleic Acids Res. 2007;35:W265–8 17485477 10.1093/nar/gkm286 PMC1933203

[70] Ou S , Jiang N. LTR_FINDER_parallel: parallelization of LTR_FINDER enabling rapid identification of long terminal repeat retrotransposons. Mob DNA. 2019;10:48 31857828 10.1186/s13100-019-0193-0 PMC6909508

[71] Ou S , Jiang N. LTR_retriever: a highly accurate and sensitive program for identification of long terminal repeat retrotransposons. Plant Physiol. 2018;176:1410–22 29233850 10.1104/pp.17.01310 PMC5813529

[72] Su W , Gu X, Peterson T. TIR-learner, a new ensemble method for TIR transposable element annotation, provides evidence for abundant new transposable elements in the maize genome. Mol Plant. 2019;12:447–60 30802553 10.1016/j.molp.2019.02.008

[73] Shi J , Liang C. Generic repeat finder: a high-sensitivity tool for genome-wide de novo repeat detection. Plant Physiol. 2019;180:1803–15 31152127 10.1104/pp.19.00386 PMC6670090

[74] Xiong W , He L, Lai J. et al. HelitronScanner uncovers a large overlooked cache of Helitron transposons in many plant genomes. Proc Natl Acad Sci U S A. 2014;111:10263–8 24982153 10.1073/pnas.1410068111 PMC4104883

[75] Zhang R-G , Li G-Y, Wang X-L. et al. TEsorter: an accurate and fast method to classify LTR-retrotransposons in plant genomes. Hortic Res. 2022;9:9 10.1093/hr/uhac017 PMC9002660 35184178

[76] Lyons E , Pedersen B, Kane J. et al. The value of nonmodel genomes and an example using SynMap within CoGe to dissect the Hexaploidy that predates the Rosids. Trop Plant Biol. 2008;1:181–90

[77] Bray NL , Pimentel H, Melsted P. et al. Near-optimal probabilistic RNA-seq quantification. Nat Biotechnol. 2016;34:525–7 27043002 10.1038/nbt.3519

[78] Jones P , Binns D, Chang H-Y. et al. InterProScan 5: genome-scale protein function classification. Bioinformatics. 2014;30:1236–40 24451626 10.1093/bioinformatics/btu031 PMC3998142

